# Systematic review with meta-analysis of mid-regional pro-adrenomedullin (MR-proadm) as a prognostic marker in Covid-19-hospitalized patients

**DOI:** 10.1080/07853890.2022.2162116

**Published:** 2023-01-06

**Authors:** Bartosz Fialek, Charles De Roquetaillade, Michal Pruc, Alla Navolokina, Francesco Chirico, Jerzy Robert Ladny, Frank William Peacock, Lukasz Szarpak

**Affiliations:** a Rheumatology Department, Marshal Józef Piłsudski Memorial Hospital, Plonsk, Poland; b Department of Anesthesiology, Burn and Critical Care, University Hospitals Saint-Louis-Lariboisière, AP-HP, Paris, France; c UMR-S 942, Institut National de la Santé et de la Recherche Médicale (INSERM), Cardiovascular Markers in Stressed Conditions (MASCOT), Paris University, Paris, France; d Research Unit, Polish Society of Disaster Medicine, Warsaw, Poland; e Department of Public health and Social Medicine, International European University, Kyiv, Ukraine; f Post-Graduate School of Occupational Health, Università Cattolica del Sacro Cuore, Rome, Italy; g Health Service Department, Italian State Police, Ministry of the Interior, Milan, Italy; h Department of Emergency Medicine, Bialystok Medical University, Bialystok, Poland; i Henry JN Taub Department of Emergency Medicine, Baylor College of Medicine Houston, Houston, TX, USA; j Institute of Outcomes Research, Maria Sklodowska-Curie Medical Academy, Warsaw, Poland; k Research Institute, Maria Sklodowska-Curie Bialystok Oncology Center, Bialystok, Poland

**Keywords:** MR-proADM, mid-regional pro-adrenomedullin, marker, COVID-19, SARS-CoV-2

## Abstract

**Background:**

Mid-regional pro-adrenomedullin (MR-proADM) is useful for risk stratification in patients with sepsis and respiratory infections. The study’s purpose was to assess the available data and determine the association between MR-proADM levels and mortality in COVID-19 participants.

**Methods:**

A comprehensive literature search of medical electronic databases was performed including PubMed, Web of Science, Scopus, Cochrane, and grey literature for relevant data published from 1 January 2020, to 20 November 2022. Mean differences (MD) with 95% confidence intervals (CI) were calculated.

**Results:**

Fourteen studies reported MR-proADM levels in survivors vs. non-survivors of COVID-19 patients. Pooled analysis showed that MR-proADM level in the survivor group was 0.841 ± 0.295 nmol/L for patients who survive COVID-19, compared to 1.692 ± 0.761 nmol/L for non-survivors (MD = −0.78; 95%CI: −0.92 to −0.64; *p* < 0.001).

**Conclusions:**

The main finding of this study is that mortality of COVID-19 is linked to MR-proADM levels, according to this meta-analysis. The use of MR-proADM might be extremely beneficial in triaging, assessing probable therapy escalation, predicting potential complications during therapy or significant clinical deterioration of patients, and avoiding admission which may not be necessary. Nevertheless, in order to confirm the obtained data, it is necessary to conduct large prospective studies that will address the potential diagnostic role of MR-proADM as a marker of COVID-19 severity.

**KEY MESSAGES**
- Severity of COVID-19 seems to be linked to MR-proADM levels and can be used as a potential marker for predicting a patient’s clinical course.
- The use of MR-proADM might be beneficial in triaging, assessing probable therapy escalation, predicting potential complications during therapy or significant clinical deterioration of patients, and avoiding admission which may not be necessary.
- For patients with COVID-19, MR-proADM may be an excellent prognostic indicator because it is a marker of endothelial function that may predict the precise impact on the equilibrium between vascular relaxation and contraction and lowers platelet aggregation inhibitors, coagulation inhibitors, and fibrinolysis activators in favor of clotting factors.

## 1. Introduction

Since 2019, the SARS-CoV-2 virus pandemic has been a global challenge for medical services in terms of patient care, patient number, and early prognosis of hospitalized patients’ conditions [1–3]. Early identification and classification are critical to starting appropriate therapy in hospitalized COVID-19 patients. For seriously ill COVID-19 patients, a shorter time to effective intervention is a crucial outcome predictor. In the treatment of sepsis, pneumonia, stroke, and myocardial infarction, time is extremely important [4–6]. A such delay might have adverse consequences. With the increasing use of intensive care units (ICU) during the COVID-19 pandemic in hospitals throughout the world, it is more vital than ever to adopt early support strategies that enhance patient outcomes. In addition to major indications such as respiratory distress and hypoxia levels the emergence of numerous triage methods has made it possible to stratify patients in the ICU [7–9]. The biomarkers evaluation is one of the tests we will do on a blood sample [10]. The use of biomarkers in diagnosis, risk assessment, and medical decision-making is widespread [11–14].

The findings of observational studies and meta-analyses have already shown us a lot of potential biomarkers, however, reliable and most accurate biomarkers for early risk assessment and treatment of COVID-19 patients have yet to be identified [11,12,15,16]. Identifying patients who are in danger of dying can help and provide more frequent surveillance and therapy intensification in a shorter timeframe.

Among the many pathophysiological mechanisms of COVID-19 symptomatology, virus-induced endothelial dysfunction is thought to play a central role, resulting in impaired vascular blood flow, increased coagulability [17] capillary leakage and edema. In this perspective, measurement of plasmatic ADM could be very useful to stratify patients based on endothelial dysfunction.

Mid Regional pro-Adrenomedullin (MR-proADM) is the precursor of bio-ADM, a calcitonin peptide like procalcitonin that belongs to the calcitonin peptide family [18,19]. Although ADM has a short half-life of 22 min, MR-proADM is more stable and accurately represents ADM levels in the blood [20].

ADM is a physiologically active substance with vasodilator, positive inotropic, diuretic, natriuretic, and bronchodilator properties. ADM also inhibits insulin, aldosterone, and adrenocorticotropic hormone secretion [21]. MR-proADM has sparked interest as a key role in the progression of deteriorating patients. In patients with community-acquired pneumonia, sepsis, heart failure, chronic kidney disease and myocardial infarction, higher MR-proADM levels have been linked to illness severity and prognosis in several investigations [22–26]. It is also worth noting that MR-proADM also plays a function in sepsis and septick shock. MR-proADM is then involved in inflammatory mediation, vascular permeability, microcirculation stability as well as microcirculation stability. All the above processes contribute to the development of organ dysfunction and failure [27,28]. MR-proADM is necessary to ensure endothelium stability, and an increase of MR-proADM level is seen as a sign of organ dysfunction. Previous research has shown that ADM levels rise in inflammatory disorders to help regulate the microcirculation, defend against endothelial hyperpermeability and that plasmatic ADM levels can be used as a measure of endothelial damage severity [29,30]. The risk assessment strategy for COVID-19 patients is crucial, and with such a high burden on hospitals, it enables early treatment and medical choices, which will save many lives by allowing patients to obtain vital medical care earlier. An example of the use of MR-proADM and its effectiveness may be its use among patients presenting with acute chest pain, where in the Global Registry of Acute Coronary Events he improved the risk classification by 41% [31].

We, therefore, investigated the evidence linking elevated MR-proADM with a poorer COVID-19 prognosis. This would have several implications since it might help clinicians staging hospitalized patients and guide future trials targeting this immune mediator.

## 2. Materials and methods

### 2.1. Search strategy

This systematic review and meta-analysis followed Preferred Reporting Items for Systematic Reviews and Meta-analyses (PRISMA) guidelines for reporting [32].

To find studies examining the prognostic value of MR-proADM in COVID-19-hospitalized patients, two reviewers (M.P. and B.F.) independently searched four major electronic databases (namely, PubMed, Web of Science, Scopus, and Cochrane Central Register of Controlled Trials) from 1 January, 2020 up to 20 November 2022. Additionally, a Google Scholar search was added to the electronic database search. For each database, a specific and effective search method was employed. We used the following searching terms: ‘MR-proADM’ OR ‘mid-regional proadrenomedullin’ AND ‘SARS-CoV-2’ OR ‘COVID-19’ OR ‘novel coronavirus’ OR ‘ncov’. A search strategy using the Medical Subject Heading and text words was used. The search strategy is listed in Supplementary Table 1. All studies were entered into the Endnote software (version X7; Thomson Reuters). Reference lists of relative articles were also reviewed. When a disagreement emerged over the selection of the literary articles, it was settled by discussion with another reviewer (L.S.).

### 2.2. Eligibility criteria

For at least one or more of the following outcomes, such as COVID-19 severity, in-hospital mortality, and major complications in COVID-19 patients, we considered original studies that report the MR-proADM levels among COVID-19 patients. ICU hospitalization, ARDS, Sepsis, AKI, VTE, and issues with the cardiovascular or nervous system were all considered as major complications. Only full-text studies in English language were included.

The exclusion criteria for the meta-analysis were as follows: (1) research incorporating data from pediatric patient; (2) research incorporating data from case reports, editorials, conference papers, reviews; (3) studies published in languages other than English.

### 2.3. Data extraction

Using a predetermined extraction form, two independent authors (B.F. and M.P.) extracted the data. Discussion with a third reviewer (L.S.), if necessary, was used to settle any potential differences on the eligibility of a specific study. Data were collected using a predefined form. The following data was taken from each study: publication data (last name of the first author, year of publication, study design), MR-proADM levels in predefined groups (i.e. survivors and non-survivors patients).

### 2.4. Quality assessment of the studies

The Newcastle-Ottawa Scale was used to assess study quality [33]. Three criteria are used by NOS to evaluate a study’s quality: selection, comparability, and exposure. These three factors each had a maximum score of 4, 2 and 3, respectively. High-quality studies were those with NOS ratings ≥7. The quality assessment was performed independently by two reviewers (B.F. and M.P.) and the disagreements were resolved by discussion with a third reviewer (L.S.).

### 2.5. Statistical analysis

The RevMan 5.4 software (Cochrane Collaboration, UK) was used for data analysis in the current meta-analysis. For continuous data, mean differences (MDs) with 95% confidence intervals (CIs) were calculated. In the case when MR-proADM levels were reported as median with interquartile range, estimated means and standard deviations with the formula described by Hozo were used [34]. *I*^2^ was used to investigate heterogeneity among studies. *I*^2^ values ≤25%, 25–50%, and ≥50%, represent respectively low, moderate, and high heterogeneity [35]. The random-effects model was used for *I*^2^ >50%; otherwise, the fixed effects model was employed. Egger’s test was used to assess the risk of publication bias. To indicate nominal statistical significance the two-sided *p* values <0.05 were used.

## 3. Results

### 3.1. Study selection

**Figure 1.**
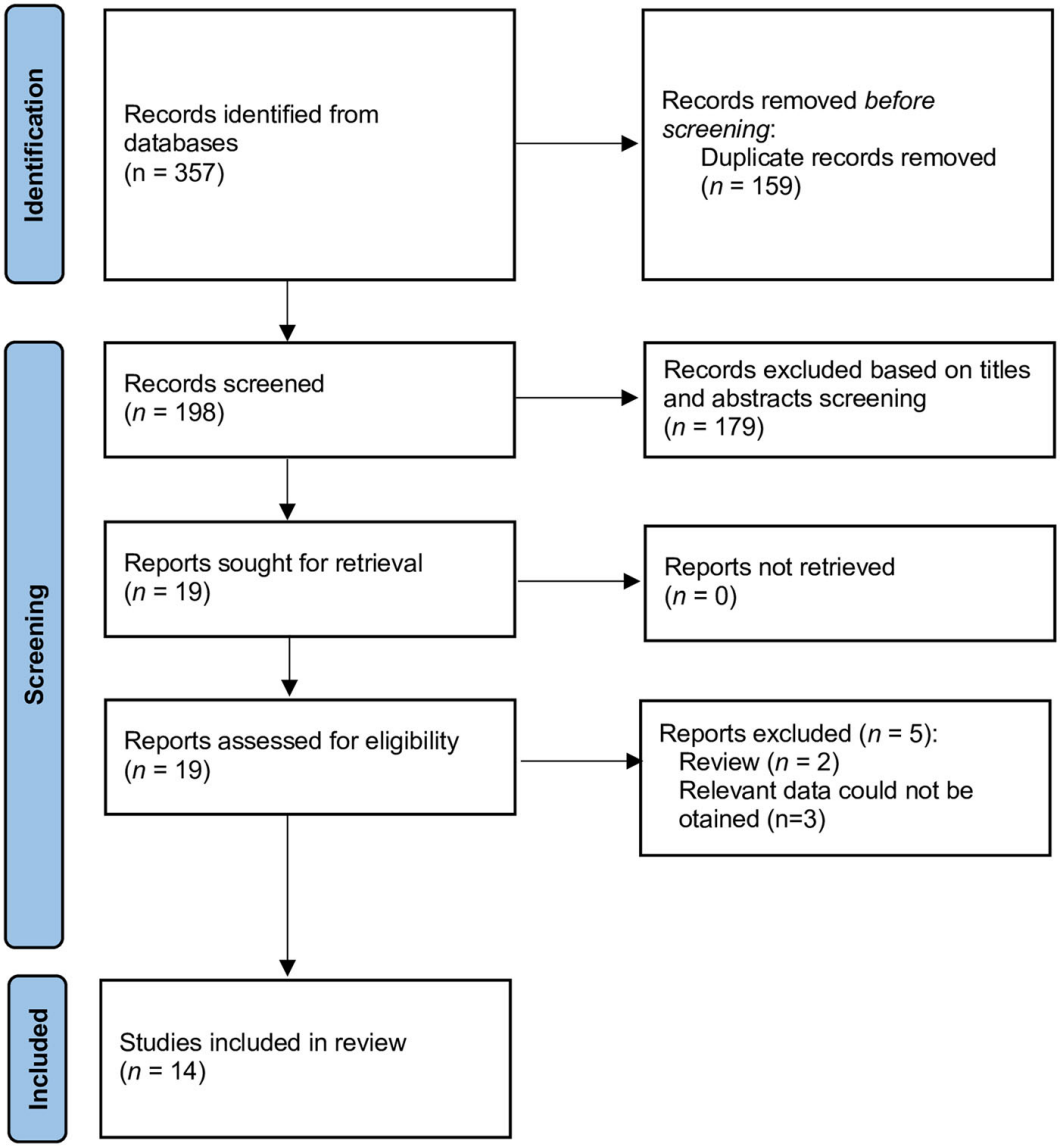
Meta-analysis flow chart of included and excluded studies.

A total of 357 articles were found after the initial search. After excluding 159 duplicates, 198 records remained for title/abstract screening. Following the screening of titles, and abstracts, the search came down to nineteen articles that have been assessed for full-text evaluation. Fourteen studies were ultimately included in the qualitative and quantitative analyses [36–49]. Figure 1 shows the flow diagram of the study selection process.

### 3.2. Study characteristics

Details for included studies are summarized in Table 1. Fourteen studies were included in the meta-analysis, with a total of 2,384 patients. Of the above studies, ten are prospective observational studies [37,38,41–44,46–49], and four are retrospective observational studies [36,39,40,45]. Five of them were conducted in Italy [36,39–41,47], two in Spain [37,42], two in Russia [44,49], one in Switzerland [38], one in the Netherlands [43], one in USA [45], one in France [46] and one in United Kingdom [48]. The NOS scores of the fourteen included studies were ≥7 (Table 1).

**Table 1.** Characteristics of included trials.

Study	Country	Study design	Survivors group						Non-survivors group						NOS score
			No. of patients	Age	Sex, male	BMI	Hypertension	Diabetes mellitus	No. of patients	Age	Sex, male	BMI	Hypertension	Diabetes mellitus	
Astapovskii et al. 2022	Russia	Prospective, observational study	110	66 (62–67)	38	NS	66 (60.0%)	14 (12.7%)	30	76 (73.2–78.2)	10	NS	23 (76.6%)	10 (33.3%)	8
Atallah et al. 2022	USA	Retrospective analysis of prospective trial	173	NS	NS	NS	NS	NS	9	NS	NS	NS	NS	NS	7
De Montmollin et al. 2022	France	Prospective, observational study	89	58.3 (49.2–66.1)	59	29.6 (26.3–34)	NS	NS	46	71.1 (62.3–75.9)	33	28.6 (24.3–31.2)	NS	NS	8
Fabris et al. 2022	Italy	Retrospective observational study	350	67.4 (55.6–77.2)	228 (65.1%)	NS	NS	NS	65	77.6 (69.4–83.7)	44 (67.7%)	NS	NS	NS	7
García de Guadiana- Romualdo et al. 2021	Spain	Prospective, observational study	327	57 (46–68)	209 (63.9%)	NS	143 (43.7%)	73 (22.3%)	32	76 (65–82)	21 (65.6%)	NS	22 (68.8%)	18 (56.3%)	8
Gregoriano et al. 2021	Switzerland	Prospective, observational study	72	63 (55.5–74.0)	42 (58.3%)	NS	39 (54.2%)	17 (23.6%)	17	74 (69–80)	16 (94.1%)	NS	13 (76.5%)	4 (23.5%)	7
Indirli et al. 2022	Italy	Retrospective observational study	95	NS	NS	NS	NS	NS	21	NS	NS	NS	NS	NS	8
Mangioni et al. 2022	Italy	Prospective, observational study	87	63 (53–73)	56	25.5 (23.4–29.4)	35 (40.2%)	NS	13	77 (73–82)	8	29.3 (27–31.9)	8 (61.5%)	NS	8
Minieri et al. 2022	Italy	Retrospective observational study	224	59.6 (14.6)	145 (64.7%)	NS	70 (31.3%)	19 (8.5%)	97	71.9 (11.2)	70 (70.2%)	NS	61 (62.9%)	23 (23.7%)	8
Montrucchio et al. 2021	Italy	Prospective, observational study	26	59 (53–67)	22 (84.6%)	NS	13 (50.0%)	2 (7.7%)	31	67 (56–74)	28 (90.3%)	NS	18 (58.1%)	9 (29.0%)	8
Moore et al. 2022	United Kingdom	Prospective, observational study	105	NS	NS	NS	NS	NS	30	NS	NS	NS	NS	NS	7
Oblitas et al. 2021	Spain	Prospective, observational study	83	58.7 ± 12.5	55 (66.3)	29 ± 4.7	NS	NS	12	71.3 ± 9.1	9 (75.0%)	29 ± 6.8	NS	NS	8
Popov et al. 2022	Russia	Prospective, observational study	115	62.2 (50.3–71.4)	NS	28.7 (25.8–32.3)	NS	NS	20	73.4 (63.5–84.8)	NS	27.6 (23.5–31.6)	NS	NS	8
van Oers et al. 2021	The Netherlands	Prospective, observational study	75	65 (58–73)	56 (74.7%)	28.3 (25.8–32.3)	20 (26.7%)	17 (22.7%)	30	72 (67–76)	24 (80.0%)	29.2 (25.5–33.3)	9 (30.0%)	7 (23.3%)	7

NOS: Newcastle Ottawa Scale; NS: not specified.

### 3.3. Meta-analysis results

**Figure 2.**
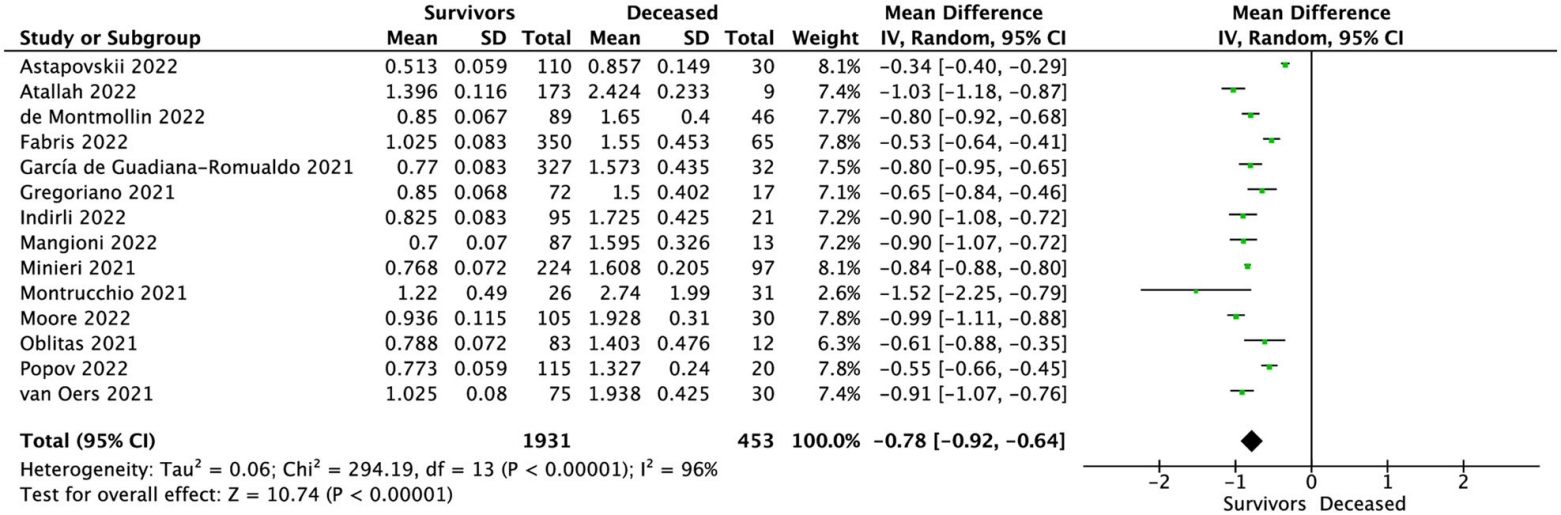
Forest plot of MR-proADM levels in survivors vs. non-survivors COVID-19 patients.

Fourteen studies reported MR-proADM levels in survivors vs. non-survivors COVID-19 patients. Pooled analysis showed that MR-proADM level in the survivor group was 0.841 ± 0.295 nmol/L for patients who survive COVID-19, compared to 1.692 ± 0.761 nmol/L for non-survivors (MD = −0.78; 95%CI: −0.92 to −0.64; *p* < 0.001; Figure 2). Sensitivity analysis based on the leave-one-out analysis showed that the pooled results were not influenced by a single trial.

Pooled analysis of two studies [39,45] showed that MR-proADM levels among patients without and with mechanical ventilation varied and amounted to 1.042 ± 0.312 vs. 1.588 ± 0.212 nmol/L, respectively (MD = −0.55; 95%CI: −0.88 to −0.22; *p* = 0.001). Additionally, study by Indirli et al. [39] ARDS, sepsis, VTE, acute kidney injury (AKI) or cardiological or neurological complications (Table 2). Analysis showed statistically significant differences in the MR-proADM levels between the groups of patients with and without the following complications: sepsis (MD = 4.22; 95%CI: 2.73–5.72; *p* < 0.001), AKI (MD = 2.52; 95%CI: 1.23–3.82; *p* < 0.001), cardiological complications (MD = 0.10; 95%CI: 0.04–0.16; *p* = 0.002) as well as in neurological complications group (MD = 0.58; 95%CI: 0.26–0.89; *p* < 0.001).

**Table 2.** Pooled analysis of MR-proADM among different patients subgroups.

Adverse event	No. of studies	MR-proADM levels		MD	95%CI	*p*-Value for differences across groups
		AE occurance group	AE non-occurance group			
Admission to ICU	1	0.975 (0.33)	0.875 (0.083)	0.10	−0.12 to 0.32	0.36
ARDS	1	0.85 (0.268)	0.925 (0.117)	−0.08	−0.22 to 0.07	0.32
Sepsis	1	5.125 (1.523)	0.9 (0.1)	4.22	2.73 to 5.72	<0.001
VTE	1	1.0 (0.268)	0.9 (0.1)	0.10	−0.10 to 0.30	0.33
AKI	1	3.425 (1.323)	0.9 (0.1)	2.52	1.23 to 3.82	<0.001
Cardiological complications	1	1.025 (0.125)	0.925 (0.117)	0.10	0.04 to 0.16	0.002
Neurological complications	1	1.475 (0.5)	0.9 (0.1)	0.58	0.26 to 0.89	<0.001

AE: adverse event; AKI: acute kidney injury; ARDS: acute respiratory distress syndrome; CI: confidence interval; ICU: intensive care unit; MD: mean difference; VTE: venous thrombotic event.

## 4. Discussion

According to current knowledge, COVID-19 is most frequently linked to respiratory system dysfunction, although the cardiovascular system is more implicated than first believed [50]. All arterial beds may thrombose as a result of endothelial homeostasis abnormalities and coagulation changes brought on by a cytokine storm in COVID-19 [51]. The equilibrium between vascular relaxation and contraction is disturbed by SARS-CoV-2. Additionally, SARS-CoV-2 raises cardiovascular risk factors through reduction of the coagulation inhibitors, platelet aggregation inhibitors and fibrinolysis activators in favor of clotting factors [52]. Because MR-proADM is a measure of endothelial function and demonstrates the precise influence on these variables, it plays a crucial role and has the potential to be an excellent prognostic indicator for COVID-19 patients. According to earlier research, MR-proADM levels have been shown to increase in inflammatory diseases, help manage microcirculation, protect against endothelial hyperpermeability, and plasmatic MR-proADM levels can be used as a measure of endothelial damage severity [29,30]. Patients who have COVID-19 in the presence of endothelial cell dysfunction may have a significant mortality risk. This is because the cytokine storm brought on by infection causes damage to the vascular endothelial cells, which intensifies the pro-thrombotic condition [53]. ADM RNA expression in whole blood is higher for COVID-19 than for other respiratory infections, and it is higher in individuals with severe illness compared to those without severe disease [54].

In this meta-analysis of fourteen studies on the prognostic role of MR-proADM in the COVID-19 patients, we tried to establish whether the incidence of MR-proADM levels was associated with higher mortality in COVID-19 patients. This is the world first meta-analysis on the role of MR-proADM as a biomarker in COVID-19 illness. MR-proADM was significantly and statistically higher among patients with negative outcome. Higher marker values may predispose patients with COVID-19 to have an unfavorable prognosis. MR-proADM is the biomarker with the strongest discriminating power for longer-term (i.e. 90-day) death in a larger Spanish cohort included COVID-19 patients hospitalized with a negative predictive value of 99.5 percent. Using MR-proADM could be recommend to identify low-risk patients who may be managed in an outpatient setting [55]. Predicting not just in-hospital mortality but also some specific complications is a novel finding using MR-proADM [39]. Long-term prediction of complications could also be used in the context of long-term complications after COVID-19 such as POST-COVID-19 syndromes or LONG-COVID-19 syndromes. Our analysis showed statistically significant differences in the MR-proADM levels between the groups of patients with and without AKI (MD = 2.52; 95%CI: 1.23 to 3.82; *p* < 0.001) - this factor could influence the prediction of both AKI and chronic kidney disease. Chronic renal failure is particularly important from a medical point of view. Confirmation of the problem of chronic renal failure among patients with COVID-19 there are results of numerous studies. According to Bower et al. among the 1.7 million persons in the population, 90,000 were COVID-19 survivors with symptoms lasting at least 30 days and it was discovered that among them, roughly 5% had an estimated glomerular filtration rate that had decreased by 30% (eGFR). Therefore, a 30% drop in eGFR was 25% more likely to occur in those with long-term COVID-19 symptoms than in non-infected individuals. It should be highlighted, though, that given how many COVID-19 patients were not hospitalized, the number of people with chronic renal failure may really be significantly higher [56]. A similar problem are also thromboembolic diseases, heart failure, stroke as well as myocarditis which risk could be predicted with MR-proADM- cardiovascular complications (MD = 0.10; 95%CI: 0.04 to 0.16; *p* = 0.002) [57]. Our pooled analysis showed statistically significant differences in the MR-proADM levels between the groups of patients with and without sepsis (MD = 4.22; 95%CI: 2.73–5.72; *p* < 0.001) when we know that MR-proADM has been described as a helpful marker for distinguishing between infection and sepsis in the setting of infectious illness [58], as well as in neurological complications group (MD = 0.58; 95%CI: 0.26–0.89; *p* < 0.001). According to past research, we may speculate that people with certain underlying clinical issues have chronically increased baseline levels of these biomarkers, or that they are more likely to experience an early, heightened systemic reaction. Our meta-analysis revealed that the studies’ presenting aspects and results varied significantly from one another. The publications used for this meta-analysis varied in a number of aspects (i.e. baseline characteristics or sample size). But we were able to demonstrate that leaving out one research did not affect the overall findings when we ran a leave-one-out sensitivity analysis to assess the stability of the results.

There are several limitations to this study. The substantial heterogeneity of the studies included in the meta-analysis, as well as the observational character of the research – retrospective analysis – are the first and most significant limitations. An internal limitation is the fact that the study protocol was not registered – however, the study protocol was approved by all investigators prior to study initiation and was not changed during the study. Another restriction might be that some drugs affect COVID-19 prognosis and alter the concentrations of circulating biomarkers. As a result, the same biomarkers should be re-evaluated considering currently available therapies. Another limitation is the fact that the studies included in the meta-analysis contain small numbers of patients.

## 5. Conclusions

The main finding of this study is that severity of COVID-19 is linked to MR-proADM levels, according to this meta-analysis. The use of MR-proADM might be beneficial in triaging, assessing probable therapy escalation, predicting potential complications during therapy or significant clinical deterioration of patients, and avoiding admission which may not be necessary. Nevertheless, in order to confirm the obtained data, it is necessary to conduct large prospective studies that will address the potential diagnostic role of MR-proADM as a marker of COVID-19 severity.

## Supplementary Material

Supplemental Material

## Data Availability

The data that support the findings of this study are available on request from the corresponding author (L.S.).
