# Supplementary material for: Systematic review with meta-analysis of mid-regional pro-adrenomedullin (MR-proadm) as a prognostic marker in Covid-19-hospitalized patients
Source: Ann Med. 2023 Jan 6;55(1):379–87. doi: 10.1080/07853890.2022.2162116 (PMC9828692; doi:10.1080/07853890.2022.2162116)
Supplement: Supplemental Material [file IANN_A_2162116_SM7385.docx]

Supplementary Text S1 – Search strategy

Medical Databases –

Limits: English Language, Publications up to November 20, 2022

| PubMed: | “MR-proADM” [All Fields] OR “mid-regional proadrenomedullin” [All Fields] OR “proADM” [All Fields] AND (“COVID-19” [All Fields] OR “COVID-19” [MeSH Terms] OR “severe acute respiratory syndrome coronavirus 2” [All Fields] OR “SARS-CoV-2” [All Fields] OR “SARS-CoV-2” [MeSH Terms] OR “2019 ncov” [All Fields] OR “2019 ncov” [All Fields] OR “ncov” [All Fields] OR (“coronavirus” [MeSH Terms] OR “coronavirus” [All Fields] OR “cov” [All Fields]) |
| --- | --- |
| EMBASE: | “MR-proADM”/exp OR “MR-proADM” OR “mid-regional proadrenomedullin”/exp OR “mid-regional proadrenomedullin” OR “proADM”/exp OR “proADM” AND (“COVID-19”/exp OR “COVID-19” OR (“severe acute respiratory syndrome coronavirus 2”/exp OR “severe acute respiratory syndrome coronavirus 2” OR (“SARS-CoV-2”/exp OR “SARS-CoV-2” OR (“ncov”/exp OR “ncov” OR (“coronavirus”/exp OR “coronavirus” OR (“cov”/exp OR “cov” OR “COVID-19”:ab OR “severe acute respiratory syndrome coronavirus 2”:ab OR “SARS-CoV-2”:ab OR “ncov”:ab OR “cov”:ab OR “coronavirus”:ab) |
| COCHRANE: | #1 (MR-proADM) or (mid-regional proadrenomedullin) or (proADM) in Clinical Trials #2 MeSH descriptor proadrenomodulin explode all trees #3 (#1 OR #2) #4 (COVID-19):ab or (severe acute respiratory syndrome coronavirus 2):ab or (SARS-CoV-2):ab or (ncov):ab or (novel coronavirus):ab in Clinical Trials #5 MeSH descriptor COVID-19 explode all trees #6 (#4 OR #5) #7 (#3 AND #6) |

Additionally, referece lists of the included trials and related reviews were manually scanned.
